# Undernutrition among Ethiopian adults living with HIV: a meta-analysis

**DOI:** 10.1186/s40795-020-00334-x

**Published:** 2020-04-16

**Authors:** Animut Alebel, Getiye Dejenu Kibret, Pammla Petrucka, Cheru Tesema, Nurilign Abebe Moges, Fasil Wagnew, Getnet Asmare, Gemechu Kumera, Zebenay Workneh Bitew, Daniel Bekele Ketema, Tesfahun Tiruneh, Mamaru Wubale Melkamu, Yitbarek Tenaw Hibstie, Belisty Temesgen, Setegn Eshetie

**Affiliations:** 1 College of Health Science, Debre Markos University, Debre Markos, Ethiopia; 2 Faculty of Health, University of Technology Sydney, Sydney, Australia; 3 College of Nursing, University of Saskatchewan, Saskatoon, Canada; 4 School of Life Sciences and Bioengineering, Nelson Mandela African Institute of Science and Technology, Arusha, Tanzania; 5 Department of Nursing, College of Health Science, Debre Tabor University, Debre Tabor, Ethiopia; 6 Department of Nursing, St. Paul’s Hospital Millennium Medical College, Addis Ababa, Ethiopia; 7 Debre Markos Referral Hospital, Debre Markos, Ethiopia; 8 College of Medicine and Health Sciences, University of Gondar, Gondar, Ethiopia

**Keywords:** HIV, Undernutrition, Adults, Ethiopia

## Abstract

**Background:**

Malnutrition and human immunodeficiency virus (HIV) are interlaced in a vicious cycle and worsened in low and middle-income countries. In Ethiopia, even though individuals are dually affected by both malnutrition and HIV, there is no a nationwide study showing the proportion of malnutrition among HIV-positive adults. Consequently, this review addressed the pooled burden of undernutrition among HIV-positive adults in Ethiopia.

**Methods:**

We searched for potentially relevant studies through manual and electronic searches. An electronic search was carried out using the database of PubMed, Google Scholar, and Google for gray literature and reference lists of previous studies. A standardized data extraction checklist was used to extract the data from each original study. STATA Version 13 statistical software was used for our analysis. Descriptive summaries were presented in tables, and the quantitative result was presented in a forest plot. Heterogeneity within the included studies was examined using the Cochrane Q test statistics and *I*^*2*^ test. Finally, a random-effects meta-analysis model was computed to estimate the pooled proportion of undernutrition among HIV-positive adults.

**Results:**

After reviewing 418 studies, 15 studies met the inclusion criteria and were included in the meta-analysis. Findings from 15 studies revealed that the pooled percentage of undernutrition among HIV-positive adults in Ethiopia was 26% (95% CI: 22, 30%). The highest percentage of undernutrition (46.8%) was reported from Jimma University specialized hospital, whereas the lowest proportion of undernutrition (12.3%) was reported from Dilla Hospital. The subgroup analyses of this study also indicated that the percentage of undernourishment among HIV-positive adults is slightly higher in the Northern and Central parts of Ethiopia (27.5%) as compared to the Southern parts of Ethiopia (25%).

**Conclusion:**

This study noted that undernutrition among HIV-positive adults in Ethiopia was quite common. This study also revealed that undernutrition is more common among HIV-positive adults with advanced disease stage, anemia, diarrhea, CD4 count less than 200 cells/mm^3^, and living in rural areas. Based on our findings, we suggested that all HIV-positive adults should be assessed for nutritional status at the time of ART commencement.

## Background

Human Immunodeficiency Virus (HIV) has become and remained a significant public health challenge, especially in developing countries, including Ethiopia. By the end of 2016, an estimated 34.5 million adults (aged 15–49 years) were living with HIV, and 1 million died due to Acquired Immunodeficiency Syndrome (AIDS) related illnesses [1]. Sub-Saharan Africa (SSA) is primarily reflected within these numbers, accounting for approximately 64% of the global HIV positive adult population [1]. Ethiopia is one of the SSA countries profoundly affected by the HIV/AIDS and related conditions despite reported prevalence dropping in the 15 to 49 years age group from 1.5% in 2011 to 1.1% in 2016 [2, 3].

Undernutrition is a common problem among peoples living with HIV, which creates a vicious cycle that may catalyze progression from HIV infection to AIDS. The relationship between HIV and malnutrition is bidirectional, with each negatively affecting the immune system [4–6], thereby resulting in malnutrition, increasing the risk of morbidity and mortality, and potentially reducing the efficacy of antiretroviral therapy (ART) [7]. Individuals, who were malnourished at the initiation of ART, experience lower survival rates compared to well-nourished counterparts [8, 9], which can be seen in instances of a relatively small departure in weight (5%) being associated with decreased survival rates [9].

Ethiopia is one of the SSA countries primarily affected by this deadly dyad. Despite significant progress in the reduction of adult malnutrition, it remains a widespread public health problem of Ethiopian adults, with 33% of Ethiopians aged 15 to 49 exhibiting undernourishment (Body mass index (BMI) < 18.5) [10]. HIV infected individuals are more prone to be malnourished than the general population. The most common reasons which predispose them for malnutrition include insufficient food intake due to loss of appetite or difficulty of swallowing, malabsorption due to diarrhea, increase metabolic demand due to advanced disease are the most common [11, 12].

In Ethiopia, individuals are dually affected by both HIV and undernutrition. As a result, the Ethiopian government acknowledged the problem and incorporated as one component of the Ethiopian HIV treatment guideline [12]. Besides to ART, people living with HIV are indicated to take supplementary foods like ready to use therapeutic food (RUTF) based on the stage of the disease [12]. New information concerning the burden of malnutrition among adults living with HIV is vital to provide quality ART care. Despite this fact, there was no nationwide study indicating the proportion of malnutrition in HIV-positive adults. Thus, this systematic review and meta-analysis aimed to estimate the pooled proportion of undernutrition among HIV-positive adults in Ethiopia. The reports obtained from this meta-analysis will serve as an input for program planners and policymakers working in the area of HIV/AIDS to assess and update the current practices to decrease the burden of undernutrition among HIV-positive adults in Ethiopia.

## Methods

### Inclusion and exclusion criteria’s

Both published and unpublished observational studies (i.e., cross-sectional, case-control, and cohort) conducted among HIV-positive adults (aged ≥15 years) in Ethiopia were included. On the other hand, studies not fully accessed after accessing abstracts were excluded after at least two email contacts with the primary author. The exclusion of these articles reflects our inability to assess the quality of articles in the absence of full text. Besides, articles reported in none-English language were excluded.

### Information sources, search, and study selection

This review was done using published and unpublished articles intended to assess the proportion of undernutrition among HIV-positive adults in Ethiopia. The studies were retrieved through manual and electronic searches. An electronic search was performed from the following databases: PubMed, Google Scholar, Cochrane Library, and Google for gray literature. In contrast, a manual search was conducted to find papers from reference lists of previous studies. The limit of the study category was human. The study included all articles published until May 1, 2018. The Preferred Reporting Items of Systematic Review and Meta-Analysis (PRISMA) guideline was used [13], and the search was conducted from the beginning of January 2017 until the end of May 2018 using the following keywords: “proportion” or “prevalence” or “burden” AND “malnutrition” OR “undernutrition” OR “malnourishment” OR “underweight” AND “HIV-positive” OR “HIV-infected” AND “Adults” AND “Ethiopia”.

### Data collection process and data items

A standardized data extraction format was used to abstract data from the included articles (**see** additional file 1), which was adapted from the Johanna Briggs Institute’s data extraction format. All relevant data for this review were extracted by two reviewers (AA and GDK). The disparities between to reviewers at the time of data abstraction were resolved through discussion. In the case of additional information or to clarify method details, the corresponding author of the original research was communicated through email. The data extraction format included primary author, study year, regions of the country where the study was conducted, study area, study design, sample size, event, response rate, and some potential biases in their methods.

### Measurement of outcome variables

The primary interest of the study was to estimate the percentage of HIV-positive adults with undernutrition (BMI < 18.5 Kg/m^2^). The p proportion of undernutrition was calculated by dividing the number of individuals having a BMI < 18.5 Kg/m^2^ to the total number of study subjects (sample size) included in the final analysis.

### Quality assessment

Two authors (AA and GDK) independently assessed the quality of each original study using the Newcastle-Ottawa Scale, a three-part approach, for cross-sectional studies quality assessment [14]. The tool has three main components. The first component graded from five stars, and mainly focused on the methodological quality of each article. The second component deals with the comparability of the study, with a possibility of two stars. The third component focused on the outcomes, and statistical analysis of each primary research and graded from three stars. Disparities between the two reviewers were resolved through discussion and disputing. At last, articles with a scale of ≥6 from 10 scales were categorized as high quality (**see** additional file 2).

### Summary measures, and synthesis of results from each primary study

Data were extracted using a standardized data format prepared in a Microsoft Excel spreadsheet and analyzed using STATA Version 13 statistical software. Since the included studies we conducted at the institution level, we used the arcsine-transformed proportion to compute the final meta-analysis. For this analysis, we used a metaprop command, which is the appropriate command for pooled proportion. Lately, the back-transformed proportions, using the arcsine variance weights for the fixed-effects model and DerSimonian-Laird weights for the random-effects model, were used to estimate pooled proportion [15].

### Heterogeneity across studies

Heterogeneity among reported proportion was assessed by computing *p*-values of Cochrane Q-statistics and *I*^2^ test [16]. In this study, significant heterogeneity was observed among the included studies (*I*^2^ = 93.47%, *p* < 0.001). As a result, a random-effects meta-analysis model was used to estimate the DerSimonian and Laird’s pooled effects.

### Additional analyses

To minimize the random variation between the primary studies, subgroup analyses was done based on different variables (i.e., geographical settings, type of hospitals, CD4 count, WHO clinical disease stage, diarrhea, residence, anemia, and dietary diversity). Additionally, a univariate meta-regression model was applied by taking sample size, publication year, and quality score of each study to investigate the sources of heterogeneity. Finally, a forest plot figure was used to present the point proportions with their 95% Cis of the primary studies.

## Results

**Fig. 1:**
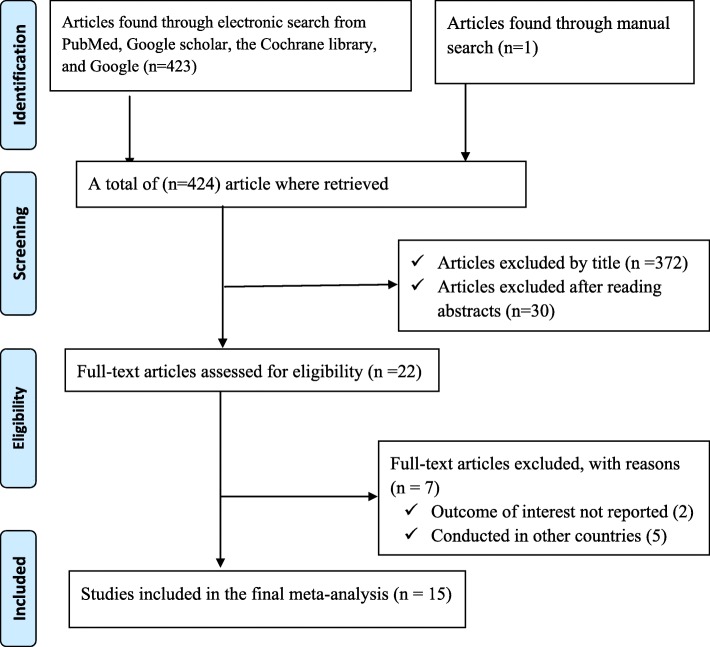
Flow chart diagrams to describe the selection of studies for a systematic review and meta-analysis of the proportion of undernutrition among HIV positive Adults in Ethiopia, 2017

During our initial search, 423 articles were collected using PubMed, Google Scholar, the Cochrane library, and Google for gray literature. Additionally, one article was obtained through a manual search from primary authors, yielding a total of 424 articles. Of these initial articles, 372 articles were excluded due to their titles being deemed irrelevant for this review. From the remaining 52 articles, 30 articles were excluded after review of their abstracts, again being assessed as non-relevant to this review. Therefore, 22 full-text articles received full consideration and were assessed for eligibility based on the pre-set criteria, which resulted in further exclusion of 7 articles, primarily due to the study locations. Concerning the reasons for excluding full articles, two articles were excluded due to our outcome of interest was not reported [17, 18]. The remaining five articles were excluded due to study location; one study each from Nigeria [19], Iran [20], Tanzania [21], Zimbabwe [22], and Botswana [23]. Finally, 15 studies met the inclusion criteria and included in the meta-analysis (Fig. 1).

### Characteristics of original studies

In the current meta-analysis, a total population of 5, 642 HIV positive adults were involved. More than half (3681 [65.2%]) were female with the mean or median age of the participants ranging from 22.4–49.9 years. With regard to study design, all included studies are cross-sectional in nature. The sample size of individual studies included in our meta-analysis ranged from 109 [24] to 719 [25]. In addition, all studies were conducted between 2006 and 2016. In the present meta-analysis, four Ethiopian regions and one administrative town were represented. Five of the studies were from Amhara [4, 26–29], three from Oromia [24, 30, 31], five from Southern Region [25, 32–35], one from Tigray [36], and one from Addis Ababa [37]. However, no studies were located in Benishangul Gumiz, Harari, or Gagmbela regions. Concerning the response rate, the majority of the included studies had a high response rate (> 85%) (Table 1).

**Table 1:** Descriptive summary of 15 studies on the proportion of undernutrition among HIV positive Adults in Ethiopia included for meta-analysis, 2006–2016

Study setting	study year	Study Design	Age	Response rate (%)	Event	Sample Size (N)	Some potential biases in their methods
Humera Hospital	2012	Cross-sectional	≥20	100	159	376	Source population and study population were not defined, outcome variable was not defined, and model fitness was not checked.
Dembia District Health Centers	2015	Cross-sectional	≥18	100	105	452	Multicollinearity was not assessed and pretest was not done.
University of Gondar Referral Hospital	2007	Cross-sectional	≥15	99.7	92	331	Multicollinearity and model fitness were not assessed.
University of Gondar Referral Hospital	2016	Cross-sectional	≥18	99.4	58	319	Training for data collectors was not given and Multicollinearity was not assessed.
Mettema Hospital	2013	Cross-sectional	≥18	99.5	131	378	Multicollinearity was not assessed.
Bahir Dar Referral Hospital	2009	Cross-sectional	≥18	100	104	408	Standardized tool was not used, outcome variable was not defined, multicollinearity was not assessed and model fitness was not checked.
Addis Ababa	2006	Cross-sectional	≥18	100	128	153	Relatively has small sample size, confounders were not controlled in the statistical analysis, standardized tool was not used, pretest was not done, and training for data collection was not given.
Jimma University specialized Hospital	2014	Cross-sectional	≥18	95.6	51	109	Relatively has small sample size, non-probable sampling technique was employed to select study participants, training for data collectors was not given, and model fitness and multicollinearity were not assessed.
Jimma University specialized Hospital	2009	Cross-sectional	≥18	100	63	319	Training for data collectors was not given, model fitness was not assessed, outcome variable was not defined, and multicollinearity was not assessed.
Nekemte Referral Hospital	2013	Cross-sectional	≥15	100	115	423	The study population and source population were not clearly defined, outcome variable was not defined, and training for data collectors was not given.
Dilla University Referral Hospital	2012	Cross-sectional	≥18	100	64	520	Inclusion and exclusion criteria were not mentioned, pretest was not done, training for data collectors was not given, and model fitness and multicollinearity were not assessed.
Butajira Hospital	2014	Cross-sectional	≥18	100	77	305	Outcome variable was not defined.
Health Facilities of Hosana Town	2014	Cross-sectional	≥18	97.1	103	330	Model fitness and multicollinearity were not assessed.
In Hawassa Health Institutions	2014	Cross-sectional	20–50	99.4	119	719	Population who are not willing to participate were excluded from the study, pregnant women were included in the study and assessed using BMI this will result bias results, and model fitness and multicollinearity were not assessed.
Wolaita Sodo Teaching and Referral Hospital	2016	Cross-sectional	≥18	96.3	133	500	Exclusion criteria was not mentioned, and model fitness and multicollinearity were not assessed.

### Quality of the included studies

The quality score of primary studies ranged from three to seven. Regarding statistical quality and data presentation, the majority of studies had poor statistical quality and data presentation methods. Besides, to identify factors associated with malnutrition, the majority [4, 24–36] of studies used binary logistic regression model for analysis, but one of the studies used Pearson correlation coefficients to determine the associations association between nutritional and immunological status [37]. Concerning the model fitness test, only two studies reported about model fitness using Hosmer and Lemeshow goodness of fit test [29, 33]. Similarly, two studies assessed multicollinearity using a VIF/tolerance [33] and the standard errors for regression coefficients [36]. Almost all studies used BMI as a measurement of nutritional status, but one study used both BMI and MUAC (mid-upper arm circumference) [24] to assess undernutrition.

### Meta-analysis

**Fig. 2:**
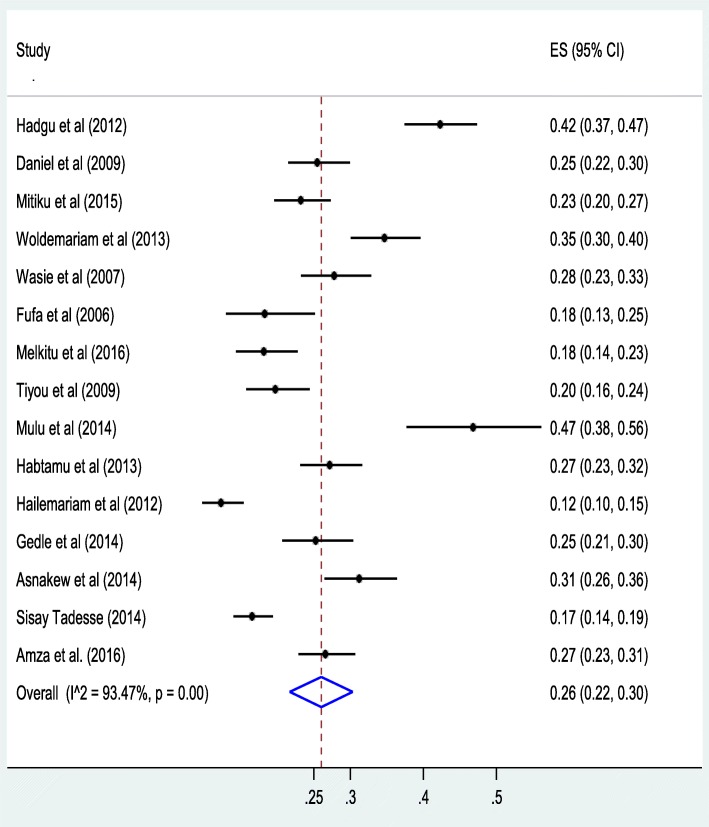
Forest plot of the pooled proportion of undernutrition among HIV positive adults in Ethiopia from 2006 to 2016

**Fig. 3:**
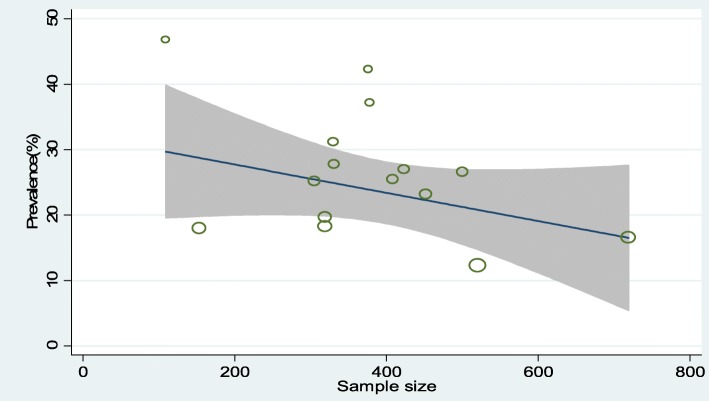
Meta-regression graph of undernutrition proportion among HIV positive adults in Ethiopia based on the sample size of the study from 2006 to 2016

The result of 15 studies revealed a pooled proportion of undernutrition among HIV-positive adults in Ethiopian was found to be 26% (95% CI: 22, 30%) (Fig. 2). In addition, the forest plot of our meta-analysis showed that the highest proportion (46.8%) of undernutrition was reported from a study done in Jimma University Specialized Hospital, Oromo Region in 2014 [24] whereas, the lowest proportion (12.3%) was reported from a study done in Dilla Hospital, Southern Region in 2012 [32]. According to the Cochrane-Q test (*p* < 0.001 and I^2^test statistics (I^2^ = 93.47%), the included studies exhibited high heterogeneity; therefore, a random-effects meta-analysis was employed in the final analysis. Besides, a univariate meta-regression analysis was conducted to identify the possible sources of heterogeneity like sample size. However, the result revealed that, as the sample size increases, the reported proportion of undernutrition in studies decreased, but the finding was not statistically significant, with a *P*-value of 0.64 (Fig. 3).

### Subgroup analyses

The subgroup proportion of undernutrition was estimated by considering different factors. These factors were: geographical settings, type of hospitals, cluster differentiation (CD4) 4 count, WHO clinical disease stage, presence of diarrhea, residence, anemia, and dietary diversity. The result of our subgroup analyses revealed that a higher proportion of undernutrition was observed among HIV-positive adults living in the Northern and central parts of Ethiopia (27%) as compared to patients living in the southern parts of Ethiopia (25%). In addition, a higher proportion of undernutrition was observed among HIV-positive patients with advanced clinical disease stage (WHO stage III and IV) (34%) as compared to milled disease stage (WHO stage I and II) (15%). Furthermore, undernutrition is more commonly prevalent among HIV-positive patients living in areas (33%) as compared to urban counterparts (22%) (Table 2).

**Table 2:** Subgroup prevalence of undernutrition among HIV positive adults in Ethiopia from 2006 to 2016 (*n* = 15)

Variables	Subgroup	No. of studies	Event	N	Proportion (95%CI)	I^2^ (%)	*P*-value
Geographical settings	Northern and Central Ethiopia	7	677	2417	27 (21, 33)	91.93	< 0.001
	Southern Ethiopia	8	725	3225	25 (19, 31)	93.70	< 0.001
Type of hospital	Referral hospitals	8	775	2785	29 (23, 34)	91.35	< 0.001
	Other than referral hospitals	7	627	2857	23 (17, 29)	93.83	< 0.001
CD4 count	< 200 cells/mm^3^	6	232	618	43 (28, 56)	93.2	< 0.001
	≥ 200 cells/mm^3^	6	360	1644	24 (14, 33)	96.1	< 0.001
WHO stage	WHO stage I and II	5	202	1366	15 (11, 18)	68.65	0.01
	WHO stage III and IV	5	375	1163	34 (18, 50)	97.25	< 0.001
Diarrhea	Yes	3	157	324	48 (43, 54)	0.00	0.82
	No	3	159	674	28 (14, 41)	93.88	< 0.001
Residence	Urban	6	370	1812	22 (16, 29)	90.81	< 0.001
	Rural	6	114	347	33 (22, 41)	72.07	< 0.001
Anemia	Yes	4	240	481	49 (41, 57)	66.84	0.03
	No	4	174	751	25 (16, 34)	85.92	< 0.001
Dietary diversity	Adequate (≥4)	3	69	451	18 (6, 30)	89.71	< 0.001
	Inadequate (< 4)	3	169	505	42 (4, 81)	99.00	< 0.001

### The time trend of undernutrition among HIV-positive adults in Ethiopia

**Fig. 4:**
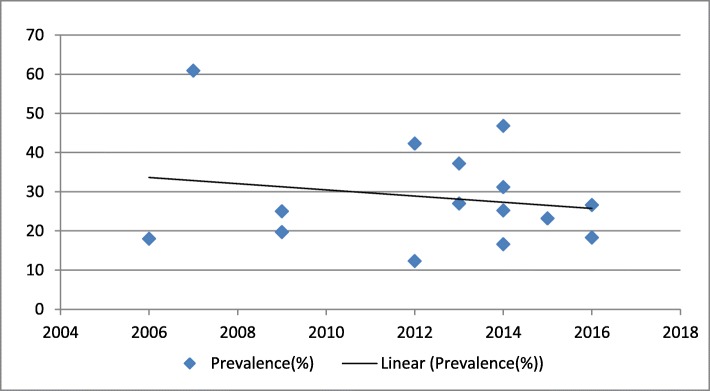
The time trend of undernutrition among HIV positive Adults in Ethiopia from 2006 to 2016

In this study, we also tried to describe the time trend of malnutrition among HIV positive adults in Ethiopia between206–2016. The result indicated that the general linear trend of undernutrition decreased in each successive year (Fig. 4).

## Discussion

Malnutrition is a global challenge. The United Nations Food and Agriculture Organization (UNFAO) estimates 795 million people in the world were suffering from chronic undernourishment in 2014–2016, with approximately 780 million living in developing countries. Within that subset, 220 million are located in Sub-Saharan Africa [38]. Further complicating and affecting the magnitude of malnutrition issues is its pervasive link to most-at-risk populations, including those living with HIV. Therefore, estimating the burden of malnutrition has a paramount contribution for clinicians as well as policymakers at various stages of HIV care to undertake corrective actions.

In Ethiopia, the proportion of undernutrition, especially among HIV positive individuals, has not been fully explored. To the best of our knowledge, this review is the first study of its kind, aimed to estimate the pooled proportion of undernutrition among HIV positive adults in Ethiopia. According to this meta-analysis, the pooled proportion of undernutrition among HIV positive adults in Ethiopia was 26% (95% CI: 22, 30%). This overall proportion is in line with a nationwide study conducted on the population of Vietnam, which suggested a proportion of 25% [39]. While recognizing the added burdens within a limited resource setting, such as sub-Saharan Africa, the current study did yield a much higher pooled proportion than some others previously conducted. For example, a meta-analysis study conducted in other SSA countries reported that the pooled proportion of HIV-related undernutrition among HIV infected women was 10.3% (95% CI: 7.4%; 14.1%) [40].

The above disparities could be due to the difference in the study area since our study conducted at the national level and gender, which would be highly suggestive of the need for a similar review within the Ethiopian context. Similarly, a nationwide study conducted among HIV-positive patients in Zimbabwe yielded a 10% proportion [22], which may reflects differences in socio-economic, cultural, and feeding pattern-related characteristics within the 2 study areas. The socio-cultural factors profoundly affect the clients’ perceived health status, response to disease, and treatment outcome [41].

Another possible explanation might be related to the study settings, since more than half (8 studies) of the studies included in this review were done at referral hospitals. From the result of our sub-group analysis, the proportion of undernutrition among studies conducted at referral hospitals was 29% (95%CI: 23, 34) whereas the proportion of undernutrition among studies other than referral hospitals was 23% (95%CI: 17, 29) (Table 2). In referral hospitals, which may indicate more advanced disease stages (Stage III and IV) and therefore increasing the risk of developing undernutrition as compared to patients within earlier disease stages. As a result, this factor might over-inflate the prevalence of undernutrition reflected. The other possible explanation might be attributed to the fact that food insecurity is epidemic in developing countries, especially in lower-income countries, which then impacts disproportionately on people living with HIV [42].

The subgroup analyses of this study revealed that a 27% proportion (95% CI: 21, 33%) of undernutrition in the northern and central parts of Ethiopia as compared to 25% within the southern regions (95% CI: 19.3, 30.7%). This discrepancy could be explained by the fact that the nutritional status of people living in Tigray and Amhara regions being impacted by land degradation and land fragmentation. These regions are traditionally the center of agriculture, which has led to soil overuse and a lack of modern inputs into agriculture. Given the current population, levels there are further challenges and, as a result, many people, especially farmers in rural areas are living under poverty. In addition, recent land redistribution in Amhara and Tigray has created fragmentation of the land, which, in turn, affects the production.

Moreover, from our sub-group analysis, we observed that undernutrition is more common among HIV-positive adults with advanced disease stage, CD4 counts less than 200 cells/mm^3^, having diarrhea, having anemia and living in rural areas. The proportion of undernutrition among HIV-positive adults with advanced disease stage was 34% (95%CI: 18, 50) which is almost two times higher as compared to the proportion of undernutrition (15% (95%CI: 11, 18)) among HIV-positive adults with mild disease stage. The biological association between disease stages and malnutrition might be due to the fact that as the disease becomes more severe, the concurrence and recurrence of opportunistic infections also become worsen.

Another finding of this review indicated that the proportion of undernutrition among HIV-positive adults with diarrhea was 48% (95%CI: 43, 54), which is higher than the proportion of undernutrition among HIV-positive adults without diarrhea, which was 28% (95%CI: 14, 41). The association between diarrhea and undernutrition is straight forward. Diarrhea increases the risk of malnutrition by reducing food appetite, energy intake, increasing nutrient loss, and decreasing nutritional absorption [43].

Furthermore, in this study, we attempted to illustrate the trend of undernutrition among HIV-positive adults in Ethiopia from 2006 to 2016. The general linear trend of undernutrition was found to be a slight decline in each successive year throughout the country (Fig. 4). The possible explanation for this trend may be nested within an area of potential reasons. For example, it might relate to the decline of adult HIV prevalence in Ethiopia from 1.5% in 2011 to 1.1% in 2016 [3, 44]. The other possible reason might be that the introduction of HAART has been associated with improved levels of undernutrition, and the ART coverage of Ethiopia was escalated for the last decade [45]. In this regard, improvements in the nutritional status of people living with HIV over a time could be due to the impact of time period because ART accessibility in Ethiopia has been escalated since 2003 (ART introduced in Ethiopia). It’s well understood that ART has a significant impact on the nutritional status of HIV-patients by reducing OIs. Another possible reason could be that the Ethiopian ART guidelines were updated in 2014 which strongly recommended initiation of ART as early as possible without any clinical or immunological criteria [46]. Furthermore, this decline might be due to since nutritional guidelines for persons living with HIV in Ethiopia which were launched in 2006 [6].

### Strengths and limitations of the study

Like other meta-analyses, this meta-analysis has many constraints that must be considered before interpreting results. Firstly, most of the studies used for this meta-analysis had small sample size. Therefore, this could have a significant effect on the estimated proportion of undernutrition. Secondly, this meta-analysis represented only studies reported from five regions and one administrative town of the country, which may yield an under-representation. Searching articles from multiple databases (both manual and electronic searches), a rigorous approach to data abstraction and analysis, as well as a clearly outlined approach were the strengths of this review.

## Conclusion

This study noted that undernutrition among HIV-positive adults in Ethiopia was quite common. This study also revealed that undernutrition is more commonly prevalent among HIV-positive adults with advanced disease stage, having anemia, having diarrhea, CD4 count less than 200 cells/mm^3^ and living in rural areas. Based on our findings, we suggested that all HIV-positive adults should assessed for nutritional status at the time of ART commencement. Moreover, a special nutritional interventions should be designed for HIV-positive adults with advanced disease stage, diarrheal diseases, anemia, and living in rural areas. Furthermore, efforts must be undertaken, which will improve the food security of people living with HIV across the nation. Lastly, further interventional studies shall be done to see the effects of disease stages, anemia, residence, and diarrhea on the treatment outcomes of undernourished-HIV positive adults.

## Supplementary information

**Additional file 1.** Data abstraction tool.

**Additional file 2.** Quality score of each study.

## Data Availability

Data will be available upon reasonable request of the corresponding author.

## Abbreviations

(CD4): cluster differentiation 4
AIDS: Acquired Immunodeficiency Syndrome
ART: Anti-retroviral Therapy
BMI: Body Mass Index
CI: Confidence Interval
HIV: Human Immunodeficiency Virus
MUAC: Mid-upper Arm Circumference
OR: Odds ratio
RUTF: Ready to Use Therapeutic Food
