# Supplementary material for: Undernutrition among Ethiopian adults living with HIV: a meta-analysis
Source: BMC Nutr. 2020 Apr 16;6:10. doi: 10.1186/s40795-020-00334-x (PMC7161140; doi:10.1186/s40795-020-00334-x)
Supplement: Supplementary file 2 — Additional file 2. Quality score of each study. [file 40795_2020_334_MOESM2_ESM.docx]

| **Table 4. Shows the quality score of each study using** Newcastle-Ottawa Scale **(NOS) quality assessment tool adapted for cross-sectional studies**. | | | | | | | | |
| --- | --- | --- | --- | --- | --- | --- | --- | --- |
| **Sample selection (maximum 5 stars)** | Wasie et al., | Fentie et al., | Hadgu et al., | Mitiku et al., | Fufa et al., | Woldemariam et al., | Mulu et al., | Daniel et al., |
| 1.**Representativeness of the sample**: Truly representative of the average in the target population. * (all subjects or random sampling), b) Somewhat representative of the average in the target population. * (non-random sampling), c) Selected group of users and d) No description of the sampling strategy | d | a* | a* | a* | a* | d | a* | d |
| 2. **Sample size**: a) **Justified and satisfactory.** * and b) Not justified. | * | * | * | * | b | b | * | b |
| 3. **Non-respondents**: a) Comparability between respondents and non-respondents characteristics is established, and the ***response rate is satisfactory.*** *, b) The response rate is unsatisfactory, or the comparability between respondents and non-respondents is unsatisfactory and c) No description of the response rate or the characteristics of the responders and the non-responders. | c | c | c | c | c | c | c | c |
| 4) **Ascertainment of the exposure (risk factor)**: a) Validated measurement tool. **, b) Non-validated measurement tool, but the tool is available or described. *, c) No description of the measurement tool. | b | c | b | b | a | b | c | b |
| **Comparability: (Maximum 2 stars)** |  |  |  |  |  |  |  |  |
| 1) The subjects in different outcome groups are comparable, based on the study design or analysis. Confounding factors are controlled. a) The study controls for the most important factor (select one). * b) The study control for any additional factor. * | b* | b* | b* | b* | b* | b* | b* | a* |
| **Outcome: (Maximum 3 stars)** |  |  |  |  |  |  |  |  |
| 1) Assessment of the outcome: a) Independent blind assessment. **, b) Record linkage. ** c) Self report. * and d) No description. | a** | a** | a** | a** | d | a** | a** | a** |
| 2) Statistical test: a) The statistical test used to analyze the data is clearly described and appropriate, and the measurement of the association is presented, including confidence intervals and the probability level (p value). * and b) The statistical test is not appropriate, not described or incomplete. | * | * | * | * | b | * | * | * |
| **Overall quality score (máximum of eight stars)** | 6 | 7 | 7 | 7 | 3 | 6 | 7 | 5 |
| **Sample selection (maximum 5 stars)** | Tiyou et al., | Asnakew et al., | Gedle et al., | Hailemariam et al., | Habtamu et al., | SisayTadesse., | Amza et al., |  |
| 1.Representativeness of the sample: Truly representative of the average in the target population. * (all subjects or random sampling), b) Somewhat representative of the average in the target population. * (non-random sampling), c) Selected group of users and d) No description of the sampling strategy | a* | a* | d | a* | a* | d | a* |  |
| 2. Sample size: a) **Justified and satisfactory.** * and b) Not justified. | b | b | * | * | b | * | * |  |
| 3. Non-respondents: a) Comparability between respondents and non-respondents characteristics is established, and the ***response rate is satisfactory.*** *, b) The response rate is unsatisfactory, or the comparability between respondents and non-respondents is unsatisfactory and c) No description of the response rate or the characteristics of the responders and the non-responders. | c | c | a* | c | c | c | c |  |
| 4) Ascertainment of the exposure (risk factor): a) Validated measurement tool. **, b) Non-validated measurement tool, but the tool is available or described. *, c) No description of the measurement tool. | b | a | a | b | b | b | c |  |
| **Comparability: (Maximum 2 stars)** |  |  |  |  |  |  |  |  |
| 1) The subjects in different outcome groups are comparable, based on the study design or analysis. Confounding factors are controlled. a) The study controls for the most important factor (select one). * b) The study control for any additional factor. * | a* | a* | b* | b* | b* | b* | b* |  |
| **Outcome: (Maximum 3 stars)** |  |  |  |  |  |  |  |  |
| 1) Assessment of the outcome: a) Independent blind assessment. **, b) Record linkage. ** c) Self report. * and d) No description. | a** | a** | a** | a** | d | a** | a** |  |
| 2) Statistical test: a) The statistical test used to analyze the data is clearly described and appropriate, and the measurement of the association is presented, including confidence intervals and the probability level (p value). * and b) The statistical test is not appropriate, not described or incomplete. | b | * | b | * | * | * | * |  |
| **Overall quality score (máximum of eight stars)** | 5 | 7 | 7 | 7 | 4 | 6 | 6 |  |

**^*^Note: NOS adapted for cross-sectional studies.**

A study can award a maximum of two stars (representing “yes”) for each item within the sample selection and outcome categories. A maximum of one star can be given for each item within comparability.
